# Procalcitonin and Presepsin as Markers of Infectious Respiratory Diseases in Children: A Scoping Review of the Literature

**DOI:** 10.3390/children11030350

**Published:** 2024-03-15

**Authors:** Giorgio Sodero, Carolina Gentili, Francesco Mariani, Valentina Pulcinelli, Piero Valentini, Danilo Buonsenso

**Affiliations:** 1 Medicine and Surgery, Catholic University of Rome, 20123 Milano, Italy; giorgio.sodero01@icatt.it (G.S.); carolina.gentili01@icatt.it (C.G.); francesco.mariani01@icatt.it (F.M.); valentina.pulcinelli01@icatt.it (V.P.); 2 Department of Woman and Child Health and Public Health, Fondazione Policlinico Universitario A. Gemelli IRCCS, 00168 Rome, Italy; piero.valentini@policlinicogemelli.it; 3 Centro di Salute Globale, Università Cattolica del Sacro Cuore, 00168 Roma, Italy

**Keywords:** procalcitonin, children, presepsin, respiratory infections

## Abstract

Introduction: Procalcitonin and presepsin have been suggested to be able to discriminate bacterial and viral infections, also in children. This scoping review aims to better explore the available evidence around the potential role of these biomarkers in the subgroup of children with respiratory infectious diseases. Methods: We performed a systematic scoping review of studies published until March 2023 in the following bibliographic databases: PubMed, EMBASE, Cochrane and SCOPUS. Results: In children with bacterial infection, procalcitonin values ranged from 0.5 ng/mL to 8.31 ng/dL, while in those hospitalized in an intensive care unit ranged from 0.6 ng/dL to 452.8 ng/dL with PCR from 2 ng/dL to 51.7 ng/dL. In children with viral infections, procalcitonin value values ranged from 0.2 ng/dL to 0.84 ng/dL, while in those hospitalized in an intensive care unit ranged from 0.61 ng/dL to 46.6 ng/dL. No studies on presepsin in children with respiratory infections were retrieved. Conclusions: Although the available literature is highly heterogeneous, evidence does not suggest a role of procalcitonin in accurately differentiating bacterial and viral infections in children with respiratory infections. In future, new approaches based on multiple markers may better help determine which febrile children require antibiotics.

## 1. Introduction

Discrimination of viral and bacterial infections in febrile children is a priority of current pediatric research as it would allow for a reduction in useless antibiotic prescriptions, therefore contributing to antibiotic stewardship programs. As clinical findings and C-Reactive Protein (CRP) have not been highly accurate in differentiating viral and bacterial infections in children [1], companies have attempted to develop new diagnostics. Procalcitonin is synthesized by tissues and organs in response to invasion by pathogenic bacteria and is increasingly being used as a marker of bacterial infections, particularly in adults, and more recently also in pediatrics [2]. Presepsin is a new marker of inflammation formed by cleavage of the N-terminal of soluble CD14, a member of the Toll-like receptor group [3]. In the last years, it has increasingly been used as an indicator of presence and severity of bacterial sepsis, although its utility in clinical practice and prognosis is not yet fully understood, particularly in children [4]. In pediatrics, it has been particularly studied for the early diagnosis of neonatal sepsis, in combination with other classic inflammation markers such as procalcitonin [5], while the role of presepsin in discriminating bacterial from viral infections in other clinical scenarios is less studied. For example, respiratory diseases still represent a major cause of mortality, morbidity and antibiotic use, and in this context, presepsin could be used as a useful discriminator of bacterial pneumonia or severity [6]. Although its use in neonates is well characterized [5], the evidence for the use of this marker in children aged >6 months is not clear because it is not a test routinely used in clinical practice and it has mainly been studied in critically ill patients or those with important comorbidities such as neutropenia [7,8], with excellent results. Both procalcitonin and presepsin have mostly been tested in critical children with suspected sepsis; however, the evidence is weaker in children with clinical symptoms suggestive of respiratory tract infections (RTIs). This is an important gap as RTIs still represent one of the commonest causes of antibiotic prescription in children [9]; therefore, understanding how newer biomarkers perform in children with RTI is a priority. This scoping review aims to analyze the use of presepsin and procalcitonin in pediatric respiratory infectious diseases, analyzing the ability to distinguish the severity and type of low respiratory tract pathology (bacterial, viral or atypical RTIs). In addition, we also attempt to compare presepsin with procalcitonin, a better-studied marker of severe bacterial infections in children. We chose a scoping review in order to first investigate the availability of literature on the topic, in order to understand current gaps and inform the potential implementation of a meta-analysis.

## 2. Materials and Methods

The study protocol has been prospectively published [10].

### 2.1. Review Questions

The main review question was “What is known about the diagnostic role of presepsin and/or PCT, either alone or in combination, in the differential diagnosis of upper and lower respiratory tract infection’s severity and etiology?”

This review also aimed assess the following sub-questions:

1. Does the adjunction of presepsin to the use of procalcitonin improve the accuracy in identifying bacterial infectious diseases?
2. What is the role of presepsin and procalcitonin in the subgroup of children with bronchiolitis?
3. What is the role of presepsin and procalcitonin in the subgroup of children with RSV bronchiolitis?

### 2.2. Inclusion Criteria

#### 2.2.1. Participants

This review included studies performed on children and adolescents (aged 0 to 17 years) with a confirmed diagnosis of upper and/or lower respiratory infectious disease (clinical, microbiological or radiological diagnosis). We included children diagnosed with pneumonia, bronchiolitis, bronchitis, croup, excluding pharyngitis, retropharyngeal abscesses, sinusitis, otitis media.

#### 2.2.2. Concept

The main concept of this review was the use of presepsin and procalcitonin in pediatric respiratory infectious diseases from different etiologies.

#### 2.2.3. Context

Considering the large spectrum of severity of the disease, we expected to find articles involving patients both hospitalized (including in the pediatric intensive care unit—PICU) or not for respiratory infections.

#### 2.2.4. Type of Sources

This review included both randomized controlled trials and non-randomized controlled trials. All the types of observational studies, prospective and retrospective (including case–control, cohort and cross-sectional studies, small case series or single case reports) have been included.

### 2.3. Search Strategy

We started our research in March 2023, without data restrictions, in the bibliographic databases PubMed, EMBASE, Cochrane and SCOPUS without date restrictions. Only articles written in English have been included. The search strategy for PubMed is available as Supplementary Materials of the published protocol [10]. The terms used for this search were adapted for use with other bibliographic databases.

### 2.4. Study Selection

After the search, the studies were exported to Rayyan. A first screening to exclude duplicates was performed by one author. Titles and/or abstracts of studies retrieved using the search strategy were screened independently by two reviewers to identify studies that could be included in this review. Full texts of potentially eligible studies were retrieved and independently assessed for eligibility by two reviewers. Each researcher was blinded to the decision of the other researcher. Any disagreement between them over the eligibility of studies was resolved through discussion and, in case of further disagreement, by discussion with a third reviewer. All the studies that did not meet the inclusion criteria were excluded. The results of the search were reported in a PRISMA flow diagram.

### 2.5. Data Extraction

Two review authors extracted data independently, each on a different Excel spreadsheet. Each researcher was blinded to the decision of the other researcher. When the process was completed, in case of discordance, any disagreement was identified and resolved through discussion (with a third author if necessary).

An Excel file was used to store data. When available, extracted information included the following:

- Study general features: title, author, year of publication, type of study, number of patients included in the study, geographical area where the study was performed;
- Participant general features: sample size of each group, nationality, age, socioeconomic status, comorbidities;
- Clinical manifestations of children included in our review;
- Main imaging findings: type of lung involvement at chest X-Ray and/or CT scan;
- Microbiological results;
- Results of the inflammation indices performed (procalcitonin and presepsin);
- Antibiotic use;
- Hospitalization, including pediatric intensive care;
- Outcomes (death, survival; survival with or without sequelae; type of sequelae).

### 2.6. Data Analysis and Presentation

To report our findings, we followed Preferred Reporting Items for Systematic reviews and Meta-Analyses extension for Scoping Reviews (PRISMA-ScR) Checklist (Supplementary Materials). We produced a narrative synthesis of the findings from the studies included in the review describing the results we obtained and providing our opinion on their interpretation. For the narrative synthesis, we preferred articles in which etiological diagnosis was specified.

### 2.7. Patient and Public Involvement

There was no direct patient or public involvement in this review.

## 3. Results

**Figure 1:**
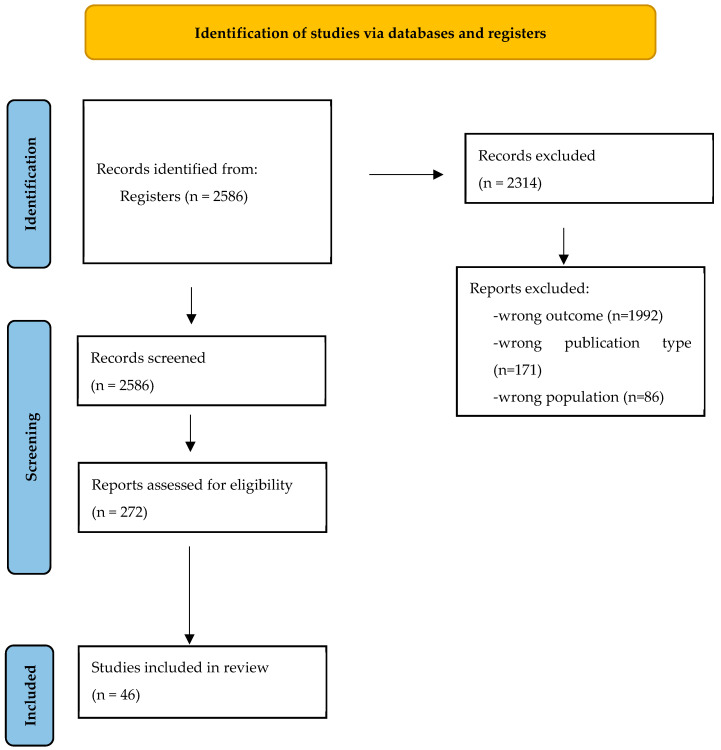
PRIMA flow for study selection. Adapted from [56].

After the preselection process, we included 45 publications in our scoping review, from a total of 28 prospective and 17 retrospective studies (Figure 1). We did not find any studies that evaluated presepsin levels in children with RTIs. The full list of studies included in this scoping review is detailed in tables, and the Excel form for all details assessed for each study is available upon request to the corresponding authors [11,12,13,14,15,16,17,18,19,20,21,22,23,24,25,26,27,28,29,30,31,32,33,34,35,36,37,38,39,40,41,42,43,44,45,46,47,48,49,50,51,52,53,54,55].

The geographical origin of the studies was heterogeneous, with most of the studies conducted in China (n = 11, 23.91%) or the United States (n = 7, 15.22%). 

**Figure 2:**
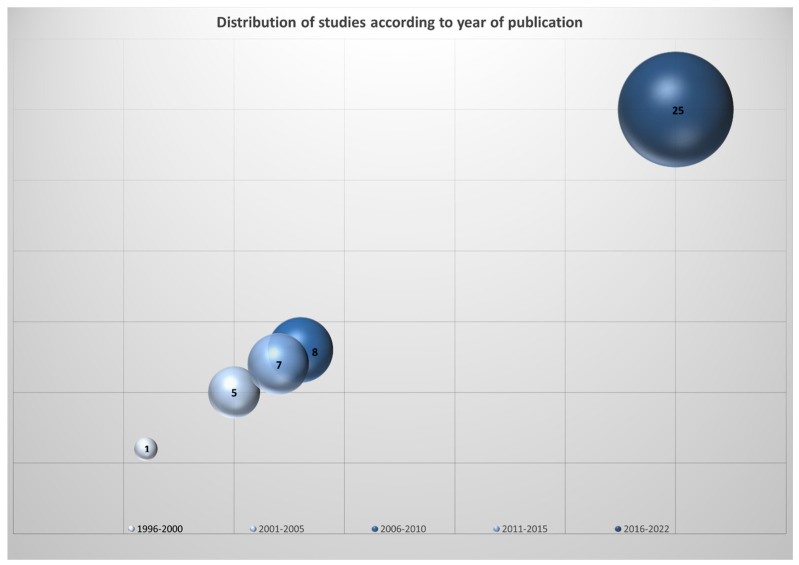
Distribution of studies according to year of publication.

The bubble chart (Figure 2) describes the distribution of studies according to year of publication: 24 studies were published between 2016 and 2022, highlighting the importance and growing interest in this type of topic; 7 were published between 2011 and 2015; 8 between 2006 and 2010; 5 between 2001 and 2005; and 1 between 1996 and 2000.

The 45 publications include a total of 30,336 pediatric patients. 

The total number of pneumonia/other lower respiratory tract infections was 22,253. Infections classified as “viral” numbered 3966 (n = 1240 RSV; n = 625 rhinovirus; n = 359 influenza virus; n = 361 adenovirus), while those classified as “bacterial” numbered 4164 (n = 640 *Streptococcus pneumoniae*; n = 3 SBEGA; n = 168 *Staphilococcus aureus*; n = 755 *Mycoplasma pneumoniae*). In the remaining cases, it was not possible to diagnose the responsible etiological agent (due to the difficulty of obtaining microbiological exams of the lower respiratory tract), although in all cases, the infective etiology was defined as probable by the treating clinicians. In three articles, the type of infection was not reported, only the severity of it. 

All descriptive information on the analyzed works is summarized in Table 1, Table 2, Table 3, Table 4 and Table 5. 

We divided the studies according to the type of infection (viral vs. bacterial) and according to the setting of the patients (regular wards vs. pediatric intensive care unit vs. both). Six studies (13.04%) analyzed patients in a PICU, while thirty-one considered patients hospitalized in a regular ward (67.39%). Nine publications recruited patients from both regular ward and ICU (19.55%). Data relating to the outcome of the patients analyzed were not collected.

As reported, children who required a more intensive care were mainly affected by Streptococcus pneumoniae, Staphylococcus aureus and Group-A Streptococcus; in those patients, PCT values and PCR values were significantly higher compared to regular setting patients. The main values of PCT and PCR are reported in Table 2. 

Among patients with a viral disease, the ones who required PICU were affected by RSV, rhinovirus, adenovirus. In those patients, PCT and PCR values were higher compared to patients in regular wards, but lower than PCT and PCR related to bacterial infections. 

Table 3 describes the main characteristics of patients with infection caused by *Mycoplasma pneumoniae*, with or without other pathogens. Most studies reported patients in regular wards; only one described patients from both settings. 

The measurement unit for procalcitonin was ng/mL, except for one study in which they used pg/mL. In patients with a bacterial infection in regular wards, PCT values ranged from 0.5 ng/mL to 8.31 ng/dL and PCR from 1 to 185.4 ng/mL; in those hospitalized in an intensive care unit, the PCT value was from 0.6 ng/dL to 452.8 ng/dL and PCR was from 2 ng/dL to 51.7 ng/dL. 

However, in viral infection and less severe infections not requiring a PICU, the PCT value was from 0.2 ng/dL to 0.84 ng/dL, and PCR was from 0.8 ng/dl to 17.32 ng/dL. In patients in an intensive care unit setting, PCT was from 0.61 ng/dL to 46.6 ng/dL, and PCR was from 1.1 ng/dL to 59 ng/dL.

Further details are provided in Table 1, Table 2, Table 3, Table 4 and Table 5.

### Synthesis of the Evidence

PCT levels are, in general, higher in children with RTIs due to bacterial infections, and in children that required PICU (in this circumstance, even in the subset of children with viral infections). However, overlap of PCT values was found in children with bacterial and viral infections, suggesting that the marker may not be extremely accurate in discriminating these categories in children with RTIs, particularly severe cases requiring PICU admission. As we did not identify any study evaluating presepsin in this type of patient, no conclusions can be obtained about the accuracy of this marker, nor optimal cutoff, for pediatric RTIs.

## 4. Discussion

We conducted a scoping review to assess whether procalcitonin and presepsin are reliable markers for differentiating viral from bacterial respiratory infections in pediatric patients. We performed this review with a scoping approach to provide a broader perspective on the role of biomarkers in pediatric practice that better translates to the complexity of daily practice, but also to provide information that may guide the development of more specific population-focused reviews.

The distinction between bacterial and viral infections based on clinical symptoms is not always straightforward [57]. Therefore, in recent years, various blood markers have been studied to improve the differential diagnosis and to enhance decision-making regarding treatment and potential antibiotic therapy [58].

In fact, antibiotics represent the most widely prescribed drugs in children worldwide and the high utilization of these drugs is leading to an increase in bacterial resistance rates, with the emergence of multidrug-resistant bacterial strains [59]. Additionally, high antibiotic consumption can cause alterations in the gut microbiota, leading to dysbiosis, reduced biodiversity and increased presence of pathogenic bacterial colonization [60], and this could potentially elevate the risk of future bacterial infections.

One of the potential therapeutic strategies is to employ personalized treatment approaches based on the patient’s clinical conditions and the results of common blood tests, to establish treatment regimens and appropriate duration [61].

CRP is one of the primary blood markers used in suspected infections; however, it is relatively non-sensitive and nonspecific, as it can be elevated in many non-infectious conditions [62], there is also no unanimous consensus on the cutoff values to be used in pediatrics for the differential diagnosis of infections. Furthermore, it has been demonstrated in pediatric populations that low CRP levels are not sufficient to rule out invasive bacterial infections [63]. Therefore, relying solely on this marker for decision-making regarding the necessity of antibiotic treatment is not advisable.

Procalcitonin, especially when assessed in conjunction with a complete blood count and CRP, exhibits better predictive values in diagnosing bacterial infections [53], but it may also increase in cases of infections caused by mycoplasma [40], mycobacteria [64] or in certain uncomplicated viral infections like influenza [65] or Sars-CoV-2 [66]; this can be explained by inflammation caused by the activation of the immune system, leading to an inflammatory response and nonspecific elevation of inflammatory indexes.

One of the more recently discovered markers is presepsin, which is a soluble CD14 subtype (sCD14-ST) [67]. Normal CD14 is a high-affinity receptor for lipopolysaccharide and is a glycoprotein expressed on the surface membranes of white blood cells, and its soluble subtype appears to increase in severe bacterial infections [68]. Among its various potential applications, presepsin has been proposed for use in neonatal sepsis diagnosis [69], although the cutoff values for its use are not yet standardized and studies evaluating its effectiveness have different formats.

Our literature analysis has highlighted that although procalcitonin maintains high accuracy in diagnosing bacterial infections, it does not possess adequate levels of sensitivity and specificity in distinguishing between pediatric respiratory infections. It may be elevated not only in pneumonia and bronchopneumonia but also in some uncomplicated viral infections.

A recent study [12] evaluated 487 children with fever and respiratory symptoms who presented to the pediatric emergency department. They underwent testing for viruses through throat swabs and routine blood analyses. The authors found that in cases of infections caused by adenovirus, an elevated procalcitonin level (cutoff used: 0.5 ng/mL) was observed in 66 out of 101 cases (52%; 16/66 with multiple viruses isolated), with even higher values in cases of multiple infections involving adenovirus and other respiratory viruses. The similar clinical presentation of bacterial respiratory infections and those caused by adenovirus does not allow for a simple differential diagnosis. Therefore, additional methods are needed for differential diagnosis and to avoid the improper use of antibiotics.

Discordant results are also reported regarding respiratory syncytial virus (RSV) infections. Hospitalized patients with RSV infections are often administered antibiotics, although the rate of serious bacterial infection and sepsis in these patients is generally low [70]. Furthermore, procalcitonin does not appear to be adequate as a marker of bacterial coinfection in patients with bronchiolitis, one of the most common RSV infections at pediatric age [47]. 

Based on these findings, it does not currently seem safe to propose a protocol based exclusively on procalcitonin to reduce antibiotic use in pediatric respiratory infections, as this marker does not consistently exhibit adequate sensitivity and specificity values. Or, at least, clinical judgment should always be taken into consideration, and results should be interpreted on the bases of other antimicrobial stewardship interventions that are of possible application in the pediatric emergency setting [71,72]. In fact, our study does not exclude a role of PCT in recognizing bacterial infections, but simply highlights that PCT results should not be interpreted as a golden rule, as they may be imperfect. In addition, these results may be translated differently in clinical practice according to the setting of application. For example, in low- to middle-income countries, where antibiotics may have a major role in reducing mortality, particularly in populations at high risk of coinfections like HIV and malaria. Even in these settings, or even more, clinical findings remain pivotal in addressing patients’ risk for more severe disease and mortality, also considering that biomarkers may not be easily available. Recently, a new Pneumonia Research Partnership to Assess WHO Recommendations (PREPARE) risk assessment tool, which includes age, sex, weight-for-age z-score, body temperature, respiratory rate, unconsciousness or decreased level of consciousness, convulsions, cyanosis and hypoxemia at baseline, has been found to have good discriminatory value when internally validated (area under the curve 0.83, 95% CI 0.81 to 0.84) for identifying children at risk of hospitalized pneumonia-related mortality [73]. In the future, validation of procalcitonin in larger populations will be necessary, as well as integration with new biomarkers based on transcriptomics, which, despite their higher costs, ref. [58] offer superior diagnostic accuracy. In addition, understanding how biomarkers can improve the accuracy of clinical prediction models like the PREPARE tool should be prioritized. Hopefully, data reporting of PCT and other biomarkers’ value should be more homogeneous to allow for comparisons between studies.

One of the possible strategies is to use existing markers in combination to increase diagnostic sensitivity. For example, MeMed BV^®^ [74] is an innovative immune-based protein signature test that measures and computationally integrates the levels of three host-proteins (TRAIL, IP-10 and CRP) to deliver fast results indicating the likelihood of a bacterial or viral infection in less than an hour, in order to optimize the use of antibiotics in cases of suspected infectious disease. Recent studies have provided promising results about its ability in discriminating bacterial from viral infections [75]. 

Surprisingly, our research did not identify any studies conducted on pediatric patients that could validate the utility of presepsin in the differential diagnosis between bacterial and viral respiratory infections. This may be due to the imperfect accuracy of our keywords and the fact that, being a new marker, studies aimed at validating its effectiveness are still being defined. Therefore, it remains a marker under validation, which currently can be used in cases of suspected sepsis.

### 4.1. Limitations

Our scoping review presents some limitations. We were unable to conduct a meta-analysis on the results of the selected articles due to their high heterogeneity (in terms of objectives, conclusions, infections and inflammatory indices considered), including about differences on how biomarkers values were reported in the different papers. In addition, the classification of respiratory infections as viral and bacterial in children has an intrinsic limitation, as bronchoalveolar lavage is rarely performed, and also, recent studies have showed that clinical value of individual pathogen detection in determining treatment is low in pediatrics [76]. In fact, most febrile children cannot be conclusively defined as having bacterial or viral infection when molecular tests supplement conventional approaches. Viruses are detected in most patients with bacterial infections. As such, we cannot exclude that some classifications of viral or bacterial infections in our review were wrong. Last, we did not update the search at time intervals to update the results. Nevertheless, this review is insightful as it highlighted that procalcitonin, although an accurate marker in diagnosing bacterial infections, does not definitively allow for a differential diagnosis of the etiology of respiratory infections in the pediatric population. Further prospective studies are needed to evaluate the sensitivity and accuracy of this marker and its ability to discriminate between bacterial and viral respiratory infections, to aid in the decision-making process for antibiotic treatment in these patients.

### 4.2. Conclusions

Procalcitonin remains an important marker in the diagnosis of serious bacterial infection. However, currently available evidence in the literature regarding procalcitonin does not document the expected role, as unique biomarker, in the differential diagnosis of pediatric respiratory infections caused by bacteria or viruses. Therefore, a multidisciplinary approach to these patients is necessary, integrating clinical objectivity and laboratory test results to determine the need for antibiotic therapy in patients with suggestive symptoms. In future, new approaches based on multiple markers may better help determine which febrile children require antibiotics.

## Figures and Tables

**Table 1:** Patients with a bacterial infection in regular wards.

Articles	N. of Patients with an Infection	Age	Sex	Comorbidities	Type of Infection	PCT Value ng/mL	PCT PPV %	PCT NPV %	PCR Value mg/L
Constanza Gómez de Oña et al. [12]	16	80 children < 1 year old, 298 between 1 and 5 years, 109 between 6 and 14.	-	-	*S. aureus*	>0.5 in 3 cases	-	-	>1 in 14 cases
Lee J.Y. et al. [18]	76	39 months (3–158 months)	M = 36 F = 40	-	*-*	2.06 ± 0.60	-	-	8.00 ± 0.75
Zhu G. et al. [20]	45	(4–7 years old)	M = 55 F = 41	-	*Streptococcus pneumoniae, S. aureus*				81.9
Pham H.T. et al. [23]	10	8.6 months	M = 19 F = 7	-	*Streptococcus pneumoniae*	1.6 (0.1–3)	83	75	5.6 (1.7–14.4)
Khan D.A. et al. [28]	46	1–12 years old	M = 28 F = 18	-	*Streptococcus pneumoniae, S. aureus*	2.69 (0.300–13.00)	-	-	6.5 (0.30–60.00)
Diez-Padrisa N. et al. [33]	89	-	M = 112 F = 64	Plasmodium Falciparum HIV	*Streptococcus pneumoniae, S. aureus*	8.31–21.75	-	-	185.4–217.4
Do Q. et al. [35]	11	5.8 (8.2)	M = 36 F = 34	-	*Streptococcus pneumoniae, S. aureus*	2.3 (0.2–4.2)	55	92	5.7 (1.7–23.6)
Korppi, M. et al. [44]	38	3 years	M = 85 F = 47	-	*Streptococcus pneumoniae,*	0.45 (0.22–1.2)	-	-	-
Hoshina T. et al. [48]	21	22 months (3–167) in bacterial and 25 (0–142) in viral group	M = 35 F = 19	Severe physical handicap and intellectual disability	*Streptococcus pneumoniae*	1.1 (0.1–13.0)	90	73	9.93 (0.12–36.69)
Erixon E.R. et al. [52]	43	2.4 years (4 days, 17 years)	M = 209 F = 165	-	-	0.25 (0.18, 056)			5.1 (4.2, 9.0)

**Table 2:** Patients with a bacterial infection in an ICU setting.

Title	N. of Patients with an Infection	Age	Sex	Comorbidities	Type of Infection	PCT Value ng/mL	PCT PPV %	PCT NPV %	PCR Value mg/L
Bashir A. et al. [11]	108	4, 62 days–17 years	M = 53 F = 55	-	*Streptococcus pneumoniae, Streptococcus pyogenes, S. aureus*	0.29 Mild–4.02 moderate/severe	−3	−4	51.7 Mild–104.8 moderate/severe
Carmina Guitart et al. [13]	97	134 days (IQR 39–554)	M = 81 F = 113	78	*Streptococcus pneumoniae, Streptococcus pyogenes, S. aureus*	0.6, 0.18, −2.26 IQR	82	64	43.1 (20–96.1 IQR)
Jullien S. et al. [14]	67	16.1 (in pneumonia group), 2–59 months	M= 84 F = 65	-	*Streptococcus pneumoniae*	452.8 (46.6–2153.2)	-	-	2.1 (1.4–4.3)
John J. et al. [15]	21	9 (1–55)	M = 49 F = 26	-	*Streptococcus pneumoniae, S. aureus*	1 ng/mL (IQR, 0.41–3.83 ng/mL)	68	76	
Zhu F. et al. [19]	34	10 months–6 years old (bacterial group) and 11 months–7 years old (non-bacterial group)	M = 16 F = 18	-	-	12.0 ± 6.7	-	-	-
Dudognon D et al. [21]	137	3.7 years (3.3) (<2–15 years)	M = 1990 F = 1839	-	*Streptococcus pneumoniae, S. aureus*	8.6 (2.7–21.6)	-	-	223 (94–316)
Page A. et al. [22]	141	13 months [IQR] 10 to 24 (from 6 to 59 months)	-	-	-	0.7 (0.3–5.2)	-	-	40.8 (16.1–126)
Pham, Hien T., et al. [31]	47	8.6 months [SD] 19.6, range: 1.0–48.7 months.	M = 121 F = 81	-	*Streptococcus pneumoniae, S. Aureus*	3.4 (5.9)	-	-	32.5 (51.8)
Stockmann C. et al. [32]	136	2.4 years; [IQR], 1.0–6.3	M = 289 F = 243	-	*-*	6.10 IQR, 0.84–22.79	17	96	-
Laham J.L. et al. [38]	15	Mean age 2 months	M = 25 F = 15	-	*Streptococcus pneumoniae, Streptococcus pyogenes*	10.4	-	-	-
Ratageri V.H. et al. [39]	370	12 months (7, 22)	M = 235 F = 135	-	-	0.1 (0.05, 0.4)	-	-	-
Zhu F. et al. [45]	34	10 months to 6 years in bacterial group; 11 months to 7 years in non-bacterial group	M = 31 F = 34	-	-	12.0 ± 6.7	-	-	-
Ericksen R.T. et al. [47]	21	4.26 (±3.72) in patients with pneumonia and 4.68 (±4.32) in patients with bronchiolitis	M = 10 F = 11	-	-	0.93 (0.25–6.64)	-	-	51.25 (21.1–107.5)
Alejandre C. et al. [51]	181	47 days (25–100.3)	M = 399 F = 276	-	*Streptococcus pneumoniae, S. Aureus*	2.7 (0.8–8.3)	76.7	86.2	39.2 (12.5–90.2)
Wang W. et al. [54]	56	3.2	M = 128 F = 136	Yes (548 various comorbidities)	-	3.95 ± 3.75			3.05 ± 2.35

**Table 3:** Patients with Mycoplasma pneumoniae infection in both settings (ICU and non).

Title	N. of Patients	Age	Sex	Comorbidities	Type of Infection	PCT Value ng/mL	PCT PPV %	PCT NPV %	PCR Value mg/L
Gan Y. et al. [17]	56	3.4 (6 months–12 years)	M = 156 F = 109	-	*Streptococcus pneumoniae, Mycoplasma*	0.25	-	-	20–39
Don, M. et al. [24]	42	3.6 years (Sixty-three percent were <5 years and 37% were 5 years old)	-	-	*Streptococcus pneumoniae, Mycoplasma*	-	-	-	-
Don, M. et al. [25]	43	3.7 years (19% were <24 months old, 43% were between 2 and 5 years and 38% were >5 years old.)	49% M	-	*Streptococcus pneumoniae, Mycoplasma*	9.43 (0.54–22.87)	57.14	-	59.5
Cheng H.-R. et al. [26]	242	64 newborns and 374 children (2 months–11 years)	274 M 174 F	-	*Mycoplasma* *S. Aureus*	1.33 ± 6.90	-	-	11.55 ± 9.31
Meyer Sauteur PM et al. [27]	63	8.6 (6.3–11.0) in mycoplasma group, 4.7 (3.9–6.2) in mycoplasma-negative group	39 M	10, not specified	*Mycoplasma* (29)	0.06 (0.04–0.14) in mycoplasma group, 0.28 (0.12–1.75) in mycoplasma negative CAP	-	-	16 (8–36) in mycoplasma group, 72 (24–170) in mycoplasma negative CAP
Schutzle H. et al. [29]	124	22 months (1 month–17 years)	189 M	-	*Mycoplasma and others not reported*	-	-	-	-
Prat, C. et al. [34]	49	Not reported (6 months–10 years)	-	-	*Streptococcus pneumoniae, Mycoplasma*	9.42 (0.078–63.32) in CAP, 0.913 (0.076–8.02) in atypical pneumonia	-	-	268 (9.62–575.8) in CAP, 66.1 (5–232.16) in atypical pneumonia
Moulin F. et al. [36]	25	2 months to 13 years	-	-	*Streptococcus pneumoniae, Mycoplasma*	10.0 (0.6–21)	96.4	60	197 (15–400)
Nascimento-Carvalho C.M. et al. [37]	48	20 months (14) (26 days–4.8 years)	M = 95 F = 64	-	*Streptococcus pneumoniae,* *Mycoplasma*	(1.47; 0.24–4.07)	52	58	-
Jiang Y. et al. [40]	152	3.67 ± 2.04	M = 95 F = 1074	-	*Mycoplasma*	0.49 ± 0.05	-	-	25.56 ± 8.25
Hatzistilianou M. et al. [41]	23	2–14 years (5.8 ± 2.9) in bacterial group, 2–14 years (6.8 ± 3.1) in viral and mycoplasma group	M = 42 F = 31	-	*Streptococcus pneumoniae,* *S. aureus, Mycoplasma*	12.63 (0.94–62.1)	93	-	3.16 (0.31–15.66)
Korppi M. et al. [42]	105	5.8 years	M = 121 F = 80	-	*Streptococcus pneumoniae,* *Mycoplasma*	-	79	-	-
Wrotek A. et al. [46]	825	29.2 months (13.9–54.8)	M = 591 F = 473	-	*Streptococcus pneumoniae,* *Streptococcus pyogenes* *S. aureus, Mycoplasma*	0.36 (0.12–1.50)	87.59	23.41	24.26 (7.67–66.94)
Korrpi M. et al. [49]	46	19 were <24 months old, 44 were 2 to 4 years old and 38 were ≥5 years old	-	-	*Mycoplasma*	-	-	-	-
Hou-Zhen F. et al. [50]	60	1.1 ± 0.3 in mycoplasma group, 1.3 ± 0.3 in control group	-	-	*Mycoplasma*	3.68 ± 1.62	-	-	14.27 ± 3.72
Li Y. et al. [53]	230	(2.84 ± 3.30)	M = 115 F = 109	-	*Streptococcus pneumoniae,* *S. aureus, Mycoplasma*	0.54 (1.56 IQR)	50.4	79.1	8.21 (IQR 29.34)
Su W. et al. [55]	106	6.9 +/− 2.1 in bacterial and 7.2 +/− 2.6 in non-bacterial pneumonia	M = 104 F = 89	-	*Streptococcus pneumoniae,* *S. aureus, Mycoplasma*	-	-	-	-
Florin, T.A et al. [16]	38	5.6 (4.6) 3 months–18 years	M = 251 F = 226	-	*S. aureus, Mycoplasma*	-	0.13 (0.09–0.19)	0.9 (0.86–0.93)	-
Toikka, P. et al. [43]	68	4.2 years old	M = 66 F = 60		*Streptococcus pneumoniae,* *Mycoplasma*	2.09	-	-	54

**Table 4:** Patients with a viral infection in a non-ICU setting.

Title	N. of Patients	Age	Sex	Comorbidities	Type of Infection	PCT Value (ng/mL)	PCR Value (mg/L)
Constanza Gómez de Oña et al. [12]	303	80 children < 1 year old, 298 between 1 and 5 years, 109 between 6 and 14.	-	-	RSV, rhinovirus, influenza, adenovirus	>0.5 in 66 adenovirus and 34 other viruses	>1 in 77 cases of adenovirus and 77 cases of other viruses
Gan Y. et al. [17]	64	3.4 (6 months–12 years)	M = 156 F = 109	-	RSV, influenza, adenovirus	0.084 (*p* = 0.208)	17.32
Lee J.Y. et al. [18]	76	39 months (3–158 months)	M = 36 F = 40	-	-	-	-
Zhu G. et al. [20]	51	(4–7 years old)	M = 55 F = 41	-	RSV, influenza, adenovirus	-	16.8
Pham H.T. et al. [23]	26	8.6 months ([SD] 9.6)	M = 19 F = 7	-	Rhinovirus	0.2 (0–0.9)	0.8 (0.3–4.7)
Don, M. et al. [24]	47	3.6 years (sixty-three percent were <5 years and 37% were 5 years old)	-	-	RSV, influenza	-	-
Don M et al. [25]	23	3.7 years (19% were <24 months old, 43% were between 2 and 5 years and 38% were >5 years old.)	49% M	-	RSV	0.53 (0.31–1.04)	Not reported
Cheng H.-R. et al. [26]	196	64 newborns and 374 children (2 months–11 years)	274 M 174 F	-	-	0.18 ± 7.10	1.84 ± 2.03
Schutzle H. et al. [29]	213	22 months (1 month–17 years)	-	-	Rhinovirus Adenovirus RSV Influenza	<0.1	-
Varpu E. et al. [30]	16	(age 0.3–8.3 years)	M = 11 F = 5	-	Adenovirus	Less than 0.5 in 14/16 patients	>40 in 12/16 patients
Diez-Padrisa N. et al. [33]	87	-	M = 112 F = 64	-	RSV, influenza, adenovirus	0.21–23.1	18.3–96.8
Prat, C. et al. [34]	34	6 months–10 years	-	-	RSV, influenza, adenovirus	0.854 (0.128–6.08)	37.35 (10.03–229.74)
Do Q. et al. [35]	59	5.8 (8.2)	M = 36 F = 34	-	RSV	0.3 (0.1–1.1)	1.5 (0.6–4.9)
Moulin F. et al. [36]	29	2 months to 13 years	-	-	RSV, influenza, adenovirus	0.63 (0.01–4.38)	39.1 (1–169)
Nascimento-Carvalho C.M. et al. [37]	57	20 months (14) (26 days–4.8 years)	M = 95 F = 64	-	RSV, rhinovirus, influenza, adenovirus	(0.65; 0.11–2.22)	-
Hatzistilianou M. et al. [41]	50	2–14 years (5.8 ± 2.9) in bacterial group, 2–14 years (6.8 ± 3.1) in viral and mycoplasma group	M = 42 F = 31	-	RSV, influenza, adenovirus	0.42 (0.1–2.13)	10.9 (1.35–32.62)
Korppi M. et al. [42]	29	5.8 years	M = 121 F = 80	-	-	-	-
Toikka, P. et al. [43]	40	4.2	M = 66 F = 60	-	RSV, rhinovirus, influenza, adenovirus	0.56	96
Korppi, M. et al. [44]	38	3 years	M = 85 F = 47	-	RSV	0.28 (0.11–0.71)	-
Wrotek A. et al. [46]	190	29.2 months (13.9–54.8)	M = 591 F = 473	-	RSV, influenza	0.22 (0.10–0.52)	7.07 (2.33–22.66)
Hoshina T. et al. [48]	10	22 months (3–167) in bacterial and 25 (0–142) in viral group	M = 35 F = 19	severe physical handicap and intellectual disability	-	0.1 (0.1–1.1)	2.11 (0.12–20.52)
Korppi M. et al. [49]	22	19 were <24 months old, 44 were 2 to 4 years old and 38 were ≥5 years old	not specified	-	-	-	-
Erixon E.R. et al. [52]	197	2.4 yr (4 days, 17 years)	M = 209 F = 165	-	-	0.14 (0.09, 0.28)	3.9 (2.5, 4.9)
Li Y. et al. [53]	116	(2.84 ± 3.30)	M = 115 F = 109	-	-	0.21 (IQR 0.44)	4.94 (IQR 10.54)
Su W. et al. [55]	87	6.9 +/− 2.1 in bacterial and 7.2 +/− 2.6 in non-bacterial pneumonia	M = 104 F = 89	-	RSV, influenza, adenovirus	-	-

**Table 5:** Patients with a viral infection in both settings (ICU and non-ICU).

Title	N. of Patients	Age	Sex	Comorbidities	Type of Infection	PCT Value (ng/mL)	PCR Value (mg/L)
Carmina Guitart et al. [13]	169	134 days (IQR 39–554)	M = 81 F = 113	Yes (78)	RSV, rhinovirus, influenza, adenovirus	-	
Jullien S. et al. [14]	89	16.1 (in pneumonia group), 2–59 months	M= 84 F = 65	-	RSV, rhinovirus, influenza, adenovirus	46.6 (46.6–253.8)	1.1 (0.4–2.9)
John J. et al. [15]	61	9 (1–55)	M = 49 F = 26	-	Rhinovirus, influenza, adenovirus	0.61 (IQR, 0.2–0.97)	-
Florin, T.A et al. [16]	248	5.6 (4.6) 3 months–18 years	M = 251 F = 226	-	RSV, rhinovirus, influenza, adenovirus	-	-
Zhu F. et al. [19]	32	10 months–6 years old (bacterial group) and 11 months–7 years old (non-bacterial group)	M = 16 F = 18	-	-	2.8 ± 1.2	
Pham, H.T et al. [31]	202	8.6 months [SD] 19.6, range: 1.0–48.7 months.	M = 121 F = 81	-	RSV, rhinovirus, influenza, adenovirus	1.1 (1.7)	12.7 (25.6)
Stockmann C. et al. [32]	349	2.4 years; [IQR], 1.0–6.3	M = 289 F = 243	-	Not reported	0.33 IQR 0.12–1.35	-
Laham J.L. et al. [38]	40	Mean age 2 months	M = 25 F = 15	-	RSV, rhinovirus	3.9 (0.2–36.3)	-
Zhu F. et al. [45]	32	10 months to 6 years in bacterial group; 11 months to 7 years in non-bacterial group	M = 31 F = 34	-	-	2.8 ± 1.2	-
Ericksen R.T. et al. [47]	35	4.26 (±3.72) in patients with pneumonia and 4.68 (±4.32) in patients with bronchiolitis	M = 10 F = 11	-	RSV, rhinovirus	1.85 (0.28–7.94)	59.0 (21.6–69.3)
Alejandre C. et al. [51]	494	47 days (25–100.3)	M = 399 F = 276	-	RSV, rhinovirus	0.2 (0.1–0.5)	11.3 (3.7–29.6)
Wang W. et al. [54]	108	3.2	M = 128 F = 136	Yes (548 various comorbidities)	-	1.07 ± 1.69	3.31 ± 1.96

## Data Availability

The data presented in this study are available on request from the corresponding author. The data are not publicly available due to local rules.

## Supplementary Materials

The following supporting information can be downloaded at: https://www.mdpi.com/article/10.3390/children11030350/s1. Table S1. PRISMA CHECKLIST ITEM. Table S2. Minimum set of information for pediatric TB studies. Figure S1. Synthesis of the evidence. Ref. [77] is cited in the Supplementary Materials.
